# Discovery of Genes Related to Insecticide Resistance in *Bactrocera dorsalis* by Functional Genomic Analysis of a *De Novo* Assembled Transcriptome

**DOI:** 10.1371/journal.pone.0040950

**Published:** 2012-08-07

**Authors:** Ju-Chun Hsu, Ting-Ying Chien, Chia-Cheng Hu, Mei-Ju May Chen, Wen-Jer Wu, Hai-Tung Feng, David S. Haymer, Chien-Yu Chen

**Affiliations:** 1 Department of Entomology, National Taiwan University, Taipei, Taiwan; 2 Research Center for Plant Medicine, National Taiwan University, Taipei, Taiwan; 3 Department of Computer Science and Information Engineering, National Taiwan University, Taipei, Taiwan; 4 Department of Bio-Industrial Mechatronics Engineering, National Taiwan University, Taipei, Taiwan; 5 Genome and Systems Biology Degree Program, National Taiwan University, Taipei, Taiwan; 6 Taiwan Agricultural Chemicals and Toxic Substances Research Institute, Council of Agriculture, Taichung, Taiwan; 7 Department of Cell and Molecular Biology, University of Hawaii at Manoa, Honolulu, Hawaii, United States of America; American University in Cairo, Egypt

## Abstract

Insecticide resistance has recently become a critical concern for control of many insect pest species. Genome sequencing and global quantization of gene expression through analysis of the transcriptome can provide useful information relevant to this challenging problem. The oriental fruit fly, *Bactrocera dorsalis*, is one of the world's most destructive agricultural pests, and recently it has been used as a target for studies of genetic mechanisms related to insecticide resistance. However, prior to this study, the molecular data available for this species was largely limited to genes identified through homology. To provide a broader pool of gene sequences of potential interest with regard to insecticide resistance, this study uses whole transcriptome analysis developed through *de novo* assembly of short reads generated by next-generation sequencing (NGS). The transcriptome of *B. dorsalis* was initially constructed using Illumina's Solexa sequencing technology. Qualified reads were assembled into contigs and potential splicing variants (isotigs). A total of 29,067 isotigs have putative homologues in the non-redundant (nr) protein database from NCBI, and 11,073 of these correspond to distinct *D. melanogaster* proteins in the RefSeq database. Approximately 5,546 isotigs contain coding sequences that are at least 80% complete and appear to represent *B. dorsalis* genes. We observed a strong correlation between the completeness of the assembled sequences and the expression intensity of the transcripts. The assembled sequences were also used to identify large numbers of genes potentially belonging to families related to insecticide resistance. A total of 90 P450-, 42 GST-and 37 COE-related genes, representing three major enzyme families involved in insecticide metabolism and resistance, were identified. In addition, 36 isotigs were discovered to contain target site sequences related to four classes of resistance genes. Identified sequence motifs were also analyzed to characterize putative polypeptide translational products and associate them with specific genes and protein functions.

## Introduction

The number of published complete or partial genomes of insect species has risen rapidly since the genome of *Drosophila melanogaster* was first reported in 2000. To date, genome sequences are available for at least 34 species of insects in the NCBI genome database, and these include a wide array of species from the orders Hemiptera, Hymenoptera, Diptera, Coleoptera and Lepidoptera. These species range from nuisance type insects such as the human louse (*Pediculus humanus*) to major medical pests such as *Anopheles gambiae* and *Aedes aegypti*.

A major category of important insect species includes those classified as agricultural pests. Some of the most devastating pests are Dipteran species in the family Tephritidae (the true fruit flies) such as the Mediterranean fruit fly (medfly, *Ceratitis capitata*) and species of the *Bactrocera*, *Rhagoletis* and *Anastrepha* genera [1]. However, for many of these important pest species, there is at present only a very limited amount of molecular information. For example, prior to this study, there were only a relatively small number of putative complete gene coding sequences for the oriental fruit fly, *Bactrocera dorsalis*, available in the NCBI database. Furthermore, most of these were identified solely on the basis of homology to genes previously characterized in other species. The lack of comprehensive molecular data severely constrains further studies of major pest species such as the oriental fruit fly, and prevents the extensive information available on the classification, ecology, reproductive behavior, and population genetics of these species from being fully utilized [2], [3].

The genus *Bactrocera* as a whole is considered to be of major significance because it includes a large number of species known to be important pests [1]. Many of these are highly invasive with high reproductive potential and wide climatic tolerance [4]. In part because of its importance, this genus has had a long history of complicated revisions in classification and taxonomy [5]. *B*. *dorsalis* is also a member of a complex that contains at least 75 closely related species, many of which are extremely difficult to distinguish using traditional methods based on morphological characters [6]. In addition, even the most useful of these morphological characters are limited to the analysis of adult stage specimens, but most infestations or biological invasions involving these species occur when material is detected at pre-adult stages in various commodities. Analyses based on DNA, which is constant throughout the life cycle, will be much more useful for this purpose.

In this regard, new molecular information based on the analysis of whole genomes, transcriptomes and proteomes will be crucial both for improved understanding of taxonomic relationships, and for identifying genes that can provide new tools and strategies for control of these pest species. In this latter area the use of insecticides and lures has been a basic strategy for the control of Tephritid flies for many years [3]. Before the 1990 s, despite some cases of extensive exposure to insecticides, Tephritid species had shown no clear evidence of resistance [7]. However, more recently, examples of the development of resistance to certain classes of insecticides have been reported for the oriental fruit fly and other Tephritids [7]. These include insecticides based on organophosphates and carbamates, and more recently, pyrethroids and spinosad [7], [8], [9].

For many of these cases, the main mechanisms of insecticide resistance involve either modification of target sites and/or enhancement of detoxification processes. In the case of target-site resistance, one of the main products subject to modification is known to be acetylcholinesterase (AChE), enzyme commission number: EC 3.1.1.8. Modification of this enzyme can lead to insensitivity toward a variety of organophosphate and carbamate based compounds [9]. In addition, similar examples of target site modifications have been documented for cases of knockdown resistance (kdr) to pyrethroids, DDT resistance through reduced sodium channel sensitivity [10], [11], resistance to spinosad and neonicotinoids through modification of the nicotinic acetylcholine receptors (nAChRs) [12], [13], [14], [15] and modification of the gamma-aminobutyric acid (GABA)-regulated chloride channel to leading to resistance to dieldrin and fipronil [16], [17].

In some species the target-site modifications have been identified through direct comparisons of gene sequences from susceptible vs. resistant lines. This includes the point mutations found in the AChE gene (*ace*) in wild type vs. resistant *B*. *dorsalis* lines [9], [18]. However, resistance phenomena may also arise from novel variants representing other genetic changes such as alternatively spliced and/or RNA edited products [12]. In these cases, detailed characterization of global differences in the transcripts produced by individuals from susceptible vs. resistant lines may be required for identification of critical genes involved in these processes.

In terms of metabolic and detoxification processes there are three groups of enzymes, the cytochrome P450 s (P450 s, EC 1.14.14.1), the carboxylesterases (COEs, EC 3.1.1.1), and the glutathione-S transferases (GSTs, EC 2.5.1.18) that have been identified as potentially being largely responsible for insecticide resistance [19]. In a range of insect species, more than one hundred members of the P450 protein family of proteins have been already been identified [20]. It is also known that the conservation of proteins in this family across species is relatively low, and efforts to identify homologs of these genes through DNA sequence similarity is often made more challenging because of the substantial differences in codon usage patterns that are known to occur when genes from *Drosophila* are compared to genes from species within the Tephritidae [21]. In addition, the understanding of these resistance mechanisms may also be complicated because multiple enzymes are often involved in the metabolism and detoxification of xenobiotics in insects.

For future research in areas such as the management of insecticide resistance, especially in species whose genomes have not been extensively characterized [22], it will be essential to have genome level molecular data for all of the different genes and enzymes actually or potentially involved in resistance phenomena [19]. This will, of necessity, also require generating transcriptome data for the analysis of genome wide gene expression profiles to circumvent the limitations on homology based studies as described above. To develop this type of data, “next-generation sequencing” (NGS) technologies (including Solexa/Illumina, SOLID/ABI, 454/Roche, and Heliscope/Helicos) that can produce massively parallel sequences in relatively short times and at considerable reductions in terms of cost and labor requirements will be used; see [23] for a brief introduction of these technologies as applied to transcriptome studies. Until relatively recently the genomes and transcriptomes of many non-model organisms had been characterized using long-read technology [2], [24], [25], [26]. However, the throughput of long-read technologies is usually much lower compared to what can be achieved using short-reads. And although the long reads (200–400 bps) are considered to be easier to assemble, they are typically obtained only at a relatively high cost. The short reads (30–100 bps), in contrast, are produced at lower cost, but can be more challenging to assemble for large and complex genomes [23].

The goals of this transcriptome study are (1) to assemble as many as possible *B*. *dorsalis* transcripts at low cost using short reads developed from Solexa/Illumina technology, (2) to use this information to make inferences about the expression profiles of genes in the genome of this species and (3) to find a broad range of genes in this species putatively involved in insecticide resistance. To achieve these goals, we generated 2G of Solexa/Illumina data to initiate the characterization of the functional genomics of *B*. *dorsalis*. The reads were first assembled *de novo*. After that, we employed the protein sequences of *D. melanogaster* to assist in identifying short transcripts that might represent the similar genes in *B*. *dorsalis*. The potential numbers of genes representing different functional groups in *B*. *dorsalis* were estimated through comparison to *D. melanogaster* genes with GO (gene ontology) annotation. The set of resultant non-redundant transcribed sequences were also annotated by alignment against known proteins, and characterized by motif matching. In the end, we identified a large number of complete and partial sequences of transcripts putatively related to insecticide resistance. This includes those related by metabolic pathways and by target-site modifications in conjunction with specific insecticides such as *ace* for organophosphates and carbamate, nAChR subunits for neonicotinoids and spinosad, the voltage gated sodium channels (VGSCs) for pyrethroids and pyrethrins, and GABA-regulated chloride channels for cyclodiene and fipronil.

## Results and Discussion

### Transcriptome assembly

The data generated by the Illumina's Solexa sequencing contained 26,111,110 reads (Table 1), each of which was approximately 90 bases (bps) [SRA accession number: SRA 048845.1]. The total length of the paired-end data was 2,349,999,900 bps. The original data set was filtered to remove unqualified reads, after which 24,068,046 reads remained. These were fed to the short-read assembler Velvet [27]. Previous studies of transcriptome assemblies have shown that when invoking Velvet, the use of multiple settings of *k* (the length of short words) is necessary because the read sampling in the transcriptome data is often not uniform [28], [29]. For this reason, we executed Velvet eleven times by setting the parameter *k* as odd numbers ranging from 21 to 41. When we set, for example, *k* = 31, Velvet generated 252,634 contigs (Table 1). After that, the program Oases was used to identify potential splicing variations as isotigs. In the end, 71,722 isotigs containing at least 100 bps of sequence were produced, belonging to 52,684 *isogroups,* each of which is a collection of isotigs that are considered as different splicing forms of a single gene. The basic statistics of the assembled sequences using *k* = 31 are shown in Table 1.

**Table 1:** Statistics of assembled sequences (k = 31).

**Raw data**		
Number of reads	26,111,110	
Total length	2,349,999,900	
**After filtering**		
Number of reads	24,068,046	
Number of paired reads	23,105,266	
Number of single reads	962,780	
Total length	2,166,124,140	
**After assembly**		
	Contig	Isotig
minimum length	61	91
maximum length	3,017	20,303
2nd-longest sequence	2,670	20,211
3rd-longest sequence	2,650	15,718
total length	34,682,487	45,110,419
avg. length	137	628
N50	159	1,152
Sequence counts:		
>0 bp	252,634	71,768
>100 bp	127,156	71,722
>500 bp	5,453	24,644
>1000 bp	533	12,225

After this *de novo* assembly, we employed the protein sequences of *D. melanogaster* to assist in the analysis of isotigs from different settings of *k*. Each set of isotigs was first aligned against the *D. melanogaster* protein sequences using blastx [30]. After alignment, each isotig was assigned to a specific *D. melanogaster* protein using the smallest E-value obtained among the alignments with length >60 amino acids and an E-value <10^−5^.

**Figure 1:**
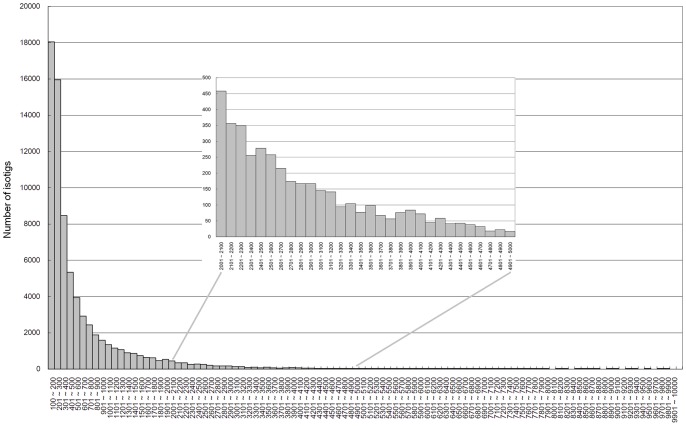
Length distribution of assembled sequences. The distribution was constructed from isotigs of length ranging from ≥100 to<10,000 base pairs.

Table 2 provides the number of distinct *D. melanogaster* RefSeq IDs that have at least one isotig from a particular *k* meeting these criteria. This number potentially refers to the number of *B*. *dorsalis* genes that have homologues in *D. melanogaster* and were discovered in this transcriptome assembly. We observed that though the numbers of distinct RefSeq IDs are similar across different *ks* (Table 2), each set of isotigs also identify transcripts that correspond to additional *D. melanogaster* proteins compared to the set where *k* = 31. For example, the setting of *k* = 21 identified the largest number (894) of additional distinct isotigs compared to the set of *k* = 31. In addition, the number of unique isotigs identified in this way decreases as the setting of *k* approaches 31. This suggests that pairs of neighboring *k*s (e.g. *k* = 29 and *k* = 31 or *k* = 31 and *k* = 33) produce more similar sets of assembled sequences when compared to pairs of *k*s with larger differences. The distinct isotigs (1,610 sequences) collected from other *k*s were also to enlarge the set of isotigs assembled by setting of *k* = 31 (71,722 sequences). In total, 73,332 isotigs were identified and used in the following analyses. The length distribution of these isotigs is plotted in Figure 1. More than 4,000 isotigs identified have a length of >2,000 bps.

**Table 2:** Results obtained using different values for *k* when invoking Velvet.

K	Number of reads not used	Number of isotigs produced	Total length (bp)	N50^a^	Number of sequences with distinct RefSeq IDs of D. melanogaster	Number of additional RefSeq IDs obtained compared to k = 31
21	11,956,988	104,334	61,744,115	1,050	9,835	894
23	12,285,614	91,507	57,017,596	1,119	9,754	783
25	12,502,653	84,639	54,220,835	1,175	9,691	647
27	12,605,728	79,320	50,553,681	1,168	9,605	555
29	12,718,986	75,216	48,128,840	1,177	9,535	390
31	13,071,912	71,722	45,106,007	1,152	9,463	0
33	13,116,734	67,967	42,511,115	1,142	9,302	299
35	13,248,990	65,040	40,173,069	1,124	9,206	378
37	13,299,322	62,193	38,338,509	1,120	9,140	453
39	13,243,018	59,279	35,908,484	1,093	8,989	503
41	13,279,423	56,388	34,039,859	1,087	8,880	519
**Union**					**11,073**	**1,610**

a The N50 length is defined as the length N for which half of all bases in the sequences are in a sequence of length L<N.

### Quality of the assembled sequences

We next used 21 complete mRNA sequences of *B. dorsalis* obtained from NCBI nucleotide database to assess the quality of the assembled data. The genes extracted from the NCBI database include the *ace* gene (accession no. AY155500). This gene sequence had earlier been obtained the same line of *B. dorsalis* used in the current study. For these assessments we required the alignment of the isotig and a known mRNA sequence to have an identity of greater than 95%. Also, the requirement for the ratio of the alignment length to the isotig length was a minimum of >80%.

Table S1 shows the results using blastn [30] to align the assembled sequences with the known *B. dorsalis* mRNA sequences. There are 14 *B. dorsalis* mRNA sequences meeting the criteria described above, including the 33 isotigs shown in Table S1. The average identity is 98.56%, which is similar to the level of sequencing accuracy reported by others for Illumina technology [31]. For six genes (GenBank accessions GU591409, HM195185, AF368053, AY155500, AY324653, and EU564816), the assembled sequences are close to the complete version of the corresponding genes. For others, however, the assembled sequences are considerably shorter.

**Figure 2:**
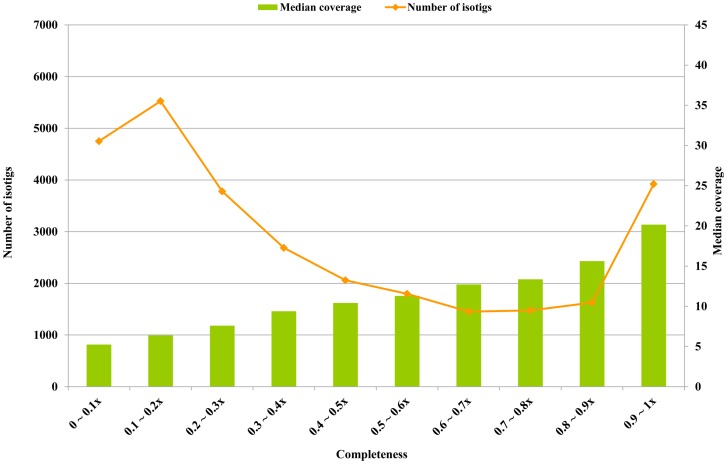
Estimated completeness of the assembled sequences. Among the assembled sequences, putative homologues were identified for 29,067 isotigs in the nr database using the requirement of a minimum alignment of length >60 amino acids and an E-value <10^−5^_._ The completeness of an isotig was estimated by the ratio of the length of the isotig versus the length of its putative homologue. Coverage represents the average depth of the isotig, where depth is defined as the total read counts observed for a particular isotig.

To make additional gene identifications the 73,332 isotigs were next aligned against the non-redundant (nr) NCBI protein database (release: January 26, 2011) using the blastx program. From this, a total of 29,067 isotigs were shown to have putative homologues in the nr database with a minimum alignment of length >60 amino acids and an E-value <10^−5^. We further examined the quality of the assembled sequences by comparing the alignment length with the sequence length of the closest homologue. We define a measure named *completeness* as the length of the assembled sequence for a particular gene versus the length of its complete form. However, since the true length of a *B. dorsalis* gene was unknown in most cases, we used the length of the nearest homologue of the isotig to estimate the complete length of the corresponding gene. The distribution of completeness values in the isotigs with these putative homologues is shown in Figure 2. By these criteria, approximately 5,546 isotigs show completeness >80% as *B. dorsalis* genes.

We also observed that there was a strong correlation between the completeness of the assembled sequences and the expression intensity of the transcripts. Figure 2 shows that the median coverage of a particular group of completed sequences consistently increases as the completeness of the sequences increases. Throughout this study, the term *coverage* stands for the average depth of an isotig, where the *depth* is defined as the read counts reported at a particular base of the isotig using the BWA program [32] and SAMtools [33]. This reflects the property of transcriptome data that transcripts with higher expression values tend to have more read counts when sequencing, and therefore have more chances to be assembled into complete transcribed sequences. From this, it is reasonable to expect that the completeness values of the assembled sequences can be improved if more read counts are generated. Finally, we further examined the assembly quality of the isotigs with lengths >10,000 bps. We observed that many of these contain a nearly complete alignment with an existing protein sequences in the nr database. Some examples are shown in Table S2.

Most of the 29,067 isotigs identified as described above using the nr database also have more than one putative homologue from different species. For this reason we further refined the identification of the isotigs with homologues in particular insects, specifically with reference to six species with complete genome data (*D. melanogaster*, *Aedes aegypti*, *Anopheles gambiae*, *Tribolium castaneum*, *Acyrthosiphon pisum,* and *Apis mellifera*). We also compared the average sequence identity of the 14,141 common isotigs across these species. Table 3 shows that the sequence similarity between *B. dorsalis* and *D. melanogaster* is the highest (72.50%). followed by *Ae. aegypti*, *An. gambiae*, *T. castaneum*, *A. mellifera*, and *A. pisum*. The result is consistent with the knowledge about the evolutionary relationships between *B. dorsalis* and these six species in FlyBase (http://flybase.org/). This also indicates that the assembled sequences are of good quality based on conserved regions.

**Table 3:** Alignments and average identities of isotigs to sequences with RefSeq IDs from other insect species.

Species	Number of *B*. *dorsalis* isotigs with homologs in other species (number of distinct protein sequences for that species in NCBI database)
*Drosophila melanogaster*	26,656 (11,808)
*Aedes aegypti*	21,467 (7,818)
*Anopheles gambiae*	21,251 (7,324)
*Tribolium castaneum*	20,734 (7,803)
*Apis mellifera*	18,495 (6,036)
*Acyrthosiphon pisum*	17,758 (6,055)
	**Average identity**
*Drosophila melanogaster*	72.50%
*Aedes aegypti*	62.62%
*Anopheles gambiae*	62.06%
*Tribolium castaneum*	58.04%
*Apis mellifera*	56.09%
*Acyrthosiphon pisum*	52.56%

Average identities were calculated based on the 14,141 common isotigs.

### Functional annotation

Since *D. melanogaster* appears be the closest model species compared *B. dorsalis*, for annotation of sequences it is most informative to find potential homologues in this species for each transcript of *B. dorsalis*. Although another well studied Tephritid species, the Mediterranean fruit fly, *Ceratitis capitata*, may be more closely related in evolutionary terms to *B. dorsalis* than either species is to *D. melanogaster* [34], the only genome level study currently available for *C. capitata* describes a collection of ESTs derived from adult head and embryonic material [2]. Incomplete characterizations of the EST sequences derived from *C. capitata* limits their use for our annotation purposes. Hence, the assembled transcripts derived here were first aligned with the available *D. melanogaster* protein sequences, and the resultant NCBI Entrez GeneIDs were sent to the software BiNGO [35]. As shown in Table 2, 11,073 *D. melanogaster* sequences were identified in this way as homologues of *B. dorsalis* transcripts. Of these, 8,815 have an NCBI Entrez GeneID.

**Figure 3:**
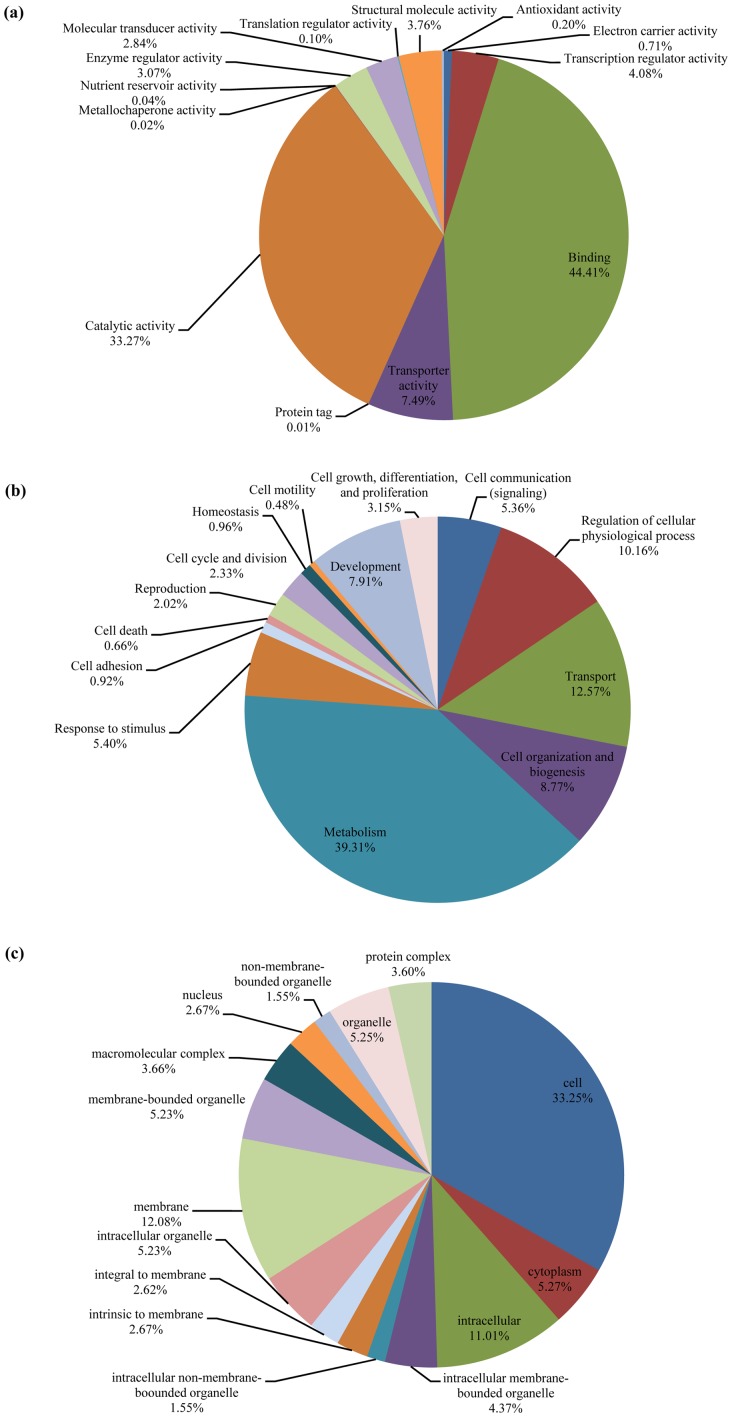
GO analysis by ontology category. **A.** Molecular function; **B.** Biological process. **C.** Cellular component.

Additional results of the BiNGO analysis are shown in Figure 3. For *B. dorsalis*, 6,007 of these sequences were associated with the 13 major GO terms representing categories of interest under ‘molecular function’, 5,387 were associated with the 14 categories of interest under ‘biological process’ and 4,991 were associated with the 15 categories of interest under ‘cellular component’. For *D. melanogaster*, by comparison, 14,904 NCBI Entrez GeneIDs were available for BiNGO analysis. Here, a larger number (8,484 GeneIDs) of these sequences were associated with the 13 major GO categories under ‘molecular function’, the 14 categories under ‘biological process’ (7,495 GeneIDs) and the 15 categories under ‘cellular component’ (7,298 GeneIDs).

To make comparisons with the *C. capitata* genome level information, we also performed an analysis aligning the 21,352 ESTs available from the NCBI database to the *D. melanogaster* protein sequences. This shows that 4,885 *D. melanogaster* sequences were identified as homologues of at least one of the *C. capitata* EST sequences and of which 4,810 had an NCBI's Entrez GeneID. After conducting the BiNGO analysis similar to that described above, 3,220 of these GeneID sequences were shown to have at least one GO term of interest under ‘molecular function’, 2,960 had terms under ‘biological process’ and 2,757 had terms under ‘cellular component’.

**Figure 4:**
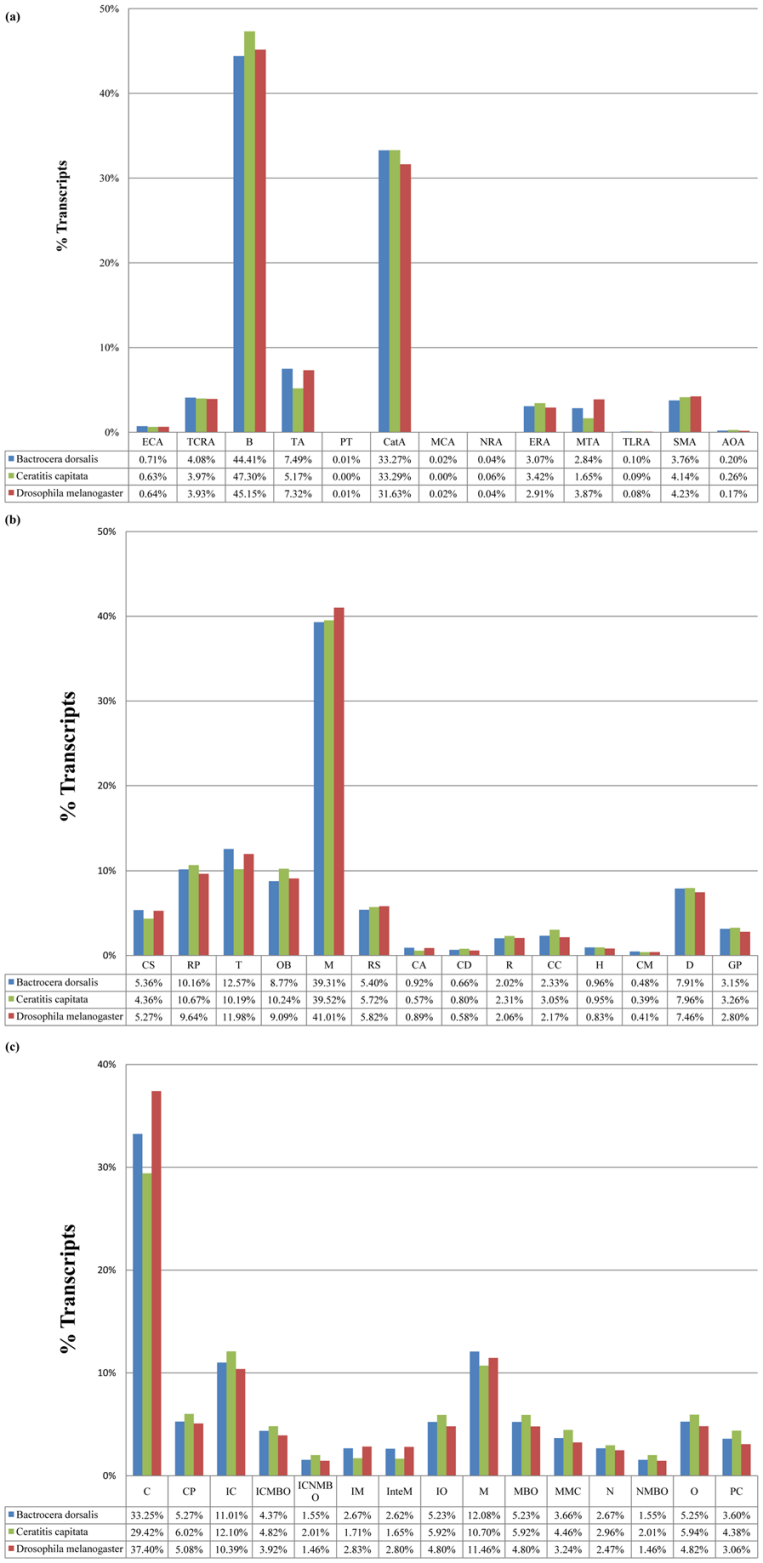
Proportions of sequences found in ontology categories for *B*. *dorsalis* compared to *C. capitata* and *D*. *melanogaster.* **A.** The 13 major categories for ontology representing ‘molecular function’ are electron carrier activity (ECA), transcription regulator activity (TCRA), binding (B), transporter activity (TA), protein tag (PT), catalytic activity (CatA), metallochaperone activity (MCA), nutrient reservoir activity (NRA), enzyme regulator activity (ERA), molecular transducer activity (MTA), translation regulator activity (TLRA), structural molecule activity (SMA), and antioxidant activity (AOA). **B.** The 14 major categories for the ontology of ‘biological process’ are cell communication (CS: signaling), regulation of cellular physiological process (RP), transport (T), cell organization and biogenesis (OB), metabolism (M), response to stimulus (RS), cell adhesion (CA), cell death (CD), reproduction (R), cell cycle and division (CC), homeostasis (H), cell motility (CM), development (D) and cell growth, differentiation, and proliferation (GP). **C.** The 15 major categories for the ontology of ‘cellular component’ are cell (C), cytoplasm (CP), intracellular (IC), intracellular membrane-bounded organelle (ICMBO), intracellular non-membrane-bound organelle (ICNMBO), intrinsic to membrane (IM), integral to membrane (InteM), intracellular organelle (IO), membrane (M), membrane-bounded organelle (MBO), macromolecular complex (MMC), nucleus (N), non-membrane-bounded organelle (NMBO), organelle (O), and protein complex (PC).

Although the actual numbers differ, overall the proportions of different sequences found in each category of ‘molecular function’, ‘biological process’ and ‘cellular component’ for *B*. *dorsalis* and *C. capitata* are similar to that of *D*. *melanogaster* (Figure 4). For example under ‘molecular function’, the category of “binding” (44.41% for *B*. *dorsalis* and 47.30% for *C. capitata*) is the largest single grouping followed by “catalytic activity” (33.27% for *B. dorsalis* and 33.29% for *C. capitata*). This is consistent with results found in many other insects [2], [24], [25]. In terms of ‘biological process’, the category of “metabolism” (39.31% for *B*. *dorsalis* and 39.52% for *C. capitata*) constituted the major portion. This was followed by “transport” as the next largest category at 12.57% in *B*. *dorsalis*. However, for *C. capitata*, the next largest group is “regulation of cellular physiological process”. In terms of ‘cellular component’, the largest single category was of “cell” as 33.25% for *B*. *dorsalis* vs. 29.42% for *C. capitata*. Similar results have been found for bedbug (*Cimex lectularius*) and whitefly (*Trialeurodes vaporariorum*) species [24], [25].

Finally, it is worthwhile to note that the data developed for virtually all of these insect studies were derived from laboratory reared strains which, depending on a number of factors such as the rearing conditions, number of generation cycles, etc., may differ to some extent in terms of genetic makeup compared to true wild flies.

### Enzyme prediction

We further characterized the putative enzymes identified based on chemical reactions catalyzed. This was achieved using predictions based on the Enzyme Commission number (EC numbers) for each assembled sequence. Assignment of isotigs into groups named with the EC numbers (four numbers separated by periods) was performed using sequence alignments from blastx against the sequenced set of enzymatic genes collected from the Uniprot database. This was followed by the characterization of these sequences using E1DS motifs [36]. Each transcript was assigned to the EC group to which the closest enzyme was found based on similarity. If more than one isotig from the same grouping had the same *D. melanogaster* protein as the nearest homologue, the one with the highest coverage was retained. The results of these predictions are provided in Table 4. For *B. dorsalis* the largest enzyme classification groups are the transferases (44.11%) followed by the hydrolases (33.01%). The rank order of the values for these two enzyme groups differs compared to that of other insect species such as the green house whitefly (*T. vaporariorum*) [25]. Otherwise, the relative proportions in other categories are in the same order and in similar ratios for these two species (Table 4).

**Table 4:** Results of enzyme predictions using EC number categories and E1DS motifs.

EC number	Name	Number/proportion of sequences predicted to be members of an enzyme group	Number of sequences matching a corresponding E1DS motif
All enzymes		6,352	1,156
1.×.×.×	Oxidoreductases	952/14.99%	307
2.×.×.×	Transferases	2,802/44.11%	348
3.×.×.×	Hydrolases	2,097/33.01%	351
4.×.×.×	Lyases	170/2.68%	40
5.×.×.×	Isomerases	160/2.52%	45
6.×.×.×	Ligases	241/3.79%	76
1.14.14.1	Cytochrome P450	115	35
2.5.1.18	Glutathione S-transferase	44	11
3.1.1.1	Carboxylesterase	42	8
3.1.1.7	Acetylcholinesterase	6	1

Genes and products representing three main types of metabolic enzymes potentially involved in insecticide resistance were also annotated through EC number predictions. These sequences are available at http://www.csbb.ntu.edu.tw/Bd/. Before releasing the assembled sequences, the isotigs reported by Velvet was confirmed by another assembler, Trinity [37], which was recently developed specifically for *de novo* transcriptome assembly. There were 13 sequences from Velvet that were assembled by Trinity into six longer sequences. In such cases, the longer ones were adopted. Two isotigs that were not confirmed by the Trinity assembly were removed. Furthermore, 49 transcripts that contain poly-N (‘N’ stands for a nucleotide position of which the base type cannot be determined) inside the assembled sequences were examined to confirm that each was respectively aligned to the same *D. melanogaster* protein sequence in the same order. Within the remaining isotigs, a total of 115 were of the P450 type (EC 1.14.14.1), 44 were of the GST type (EC 2.5.1.18), 42 were of the COE types (EC 3.1.1.1) and 6 were of the AChE related type (EC 3.1.1.7). The potential sequence names given to each of these are listed in Table S3, Table S4, Table S5, and Table S6, respectively.

For each EC group, we further employed the corresponding E1DS motifs to investigate whether the predicted sequences contained conserved motifs within each EC group. The E1DS motifs were identified using “motif discovery” on the enzyme sequences catalyzing the same chemical reactions. Our previous studies have shown that the derived motifs usually contain the essential sequence patterns associated with the catalytic sites [36], [38]. In other words, the matching of one of the E1DS motifs suggests that the assembled sequences contain the essential signatures of that EC group. As listed in Table 4, a total of 35 P450-, 11 GST-, 8 COE- and 1 AChE-related isotigs were shown to contain matches to one of the E1DS motifs. Not all the predicted transcripts contain the conserved signatures of a particular EC group, but this might be due to incompleteness of some of the assembled transcripts.

Figure S1 provides an example of the use of the EIDS motifs. The motif ‘PxLxD-x(7,10)-SxAIxxYLxxK’ was derived using motif discovery on the protein sequences belonging to EC 2.5.1.18. This motif consists of two conserved components ‘PxLxD’ and ‘SxAIxxYLxxK’, interleaved by a flexible region represented by ‘x(7,10)’. The motif components are highlighted on a 3D structure (PDB ID: 2IMK) in Figure S1 using the *sticks* presentation. This structure is of an insect Epsilon-class Glutathione S-transferase from the Malaria Vector *An. gambiae*, which shows evidence for high DDT-detoxifying activity [39]. The motif components ‘PxLxD’ and ‘SxAIxxYLxxK’ are shown to participate in the interaction with the ligand S-HEXYLGLUTATHIONE (GTX). In particular, the amino acids PRO56, SER68, and HIS69 have close contacts to the ligand GTX. In our study, six assembled sequences were identified containing this motif.

Table 5 also shows that we further examined whether the predicted isotigs containing well-known short motifs were associated with specific catalytic functions. We observed that almost all the identified sequences matching the E1DS motifs contain these motifs. This shows the potential of using E1DS motifs in characterizing assembled sequences in transcriptome studies. However, even though the motifs found here are relatively short, it is clear that not all the predicted sequences contain them. As before, the incompleteness of some of assembled transcripts might explain this situation.

**Table 5:** Presence of well-known short motifs related to specific EC groups within predicted isotigs.

EC groups	Annotated motifs (from literature)	Number of sequences with motifs/Number of sequences predicted
EC 1.14.14.1	FxxGxRxCxG	53/115
EC 2.5.1.18	SxAI	22/44
	TxAI	4/44
EC 3.1.1.1	GxSxG	26/42
EC 3.1.1.7	SEDCL	2/6

Among the isotigs shown to contain sequences related to metabolic enzymes, some correspond to the same genes. In these cases the transcripts were advanced to a filter to identify different isoforms and transcripts based on non-overlapping alignments on the reference gene pertaining to the same gene. As shown in Table 6, using this approach a total of 90 P450-, 42 GST-, 37 COE- and 1 AChE-related gene were identified in *B. dorsalis*.

**Table 6:** Number of putative genes encoding three main classes of metabolic enzymes in *B*. *dorsalis*, their families and the number of isotigs containing them.

Metabolic enzyme group	Family	Number of isotigs	Number of genes
P450 (clade)		115	90
Cyp2	Cyp304/305/307	3/4/1	2/3/1
Mitochondrial	Cyp12/49	3/1	3/1
Cyp3	Cyp6/9/28/309/310/317	51/5/4/3/1/2	37/4/4/3/1/1
Cyp4	Cyp4/311/313/318	28/3/5/1	22/2/5/1
GST		44	42
	Delta	15	14
	Epsilon	7	7
	Delta/Epsilon	6	6
	Omega	6	6
	Sigma	2	1
	Theta	3	3
	Microsomal	4	4
	unknown	1	1
COE/Cholinesterase (class)		42/6	37/1
Dietary	Alpha esterase	24	19
Pheromone and hormone processing	JHE	3	1
Neurodevelopmental	Glutactin/AChE/giotactin/neurotactin	1/1/2/1	1/1/1/1
	Unclassified	16	14

In terms of comparative genome analyses, there are some similarities and some differences for the numbers of the three main detoxification enzymes known to be involved in insecticide resistance. For example, the number of GSTs in *B. dorsalis* was 42. This number is just outside of the range of 10 to 38 genes found for *A. mellifera* and *D*. *melanogaster* [40], [41]. The number of COE transcripts was 37 in *B. dorsalis*, and this is well within the range of 24 to 51 genes seen in the *An. gambiae and A. mellifera* genomes [41]. Finally, the number of P450 genes in *B. dorsalis* (90 genes) is also within the range of 36∼180 reported for other insect species. Among this wide range in insects, the smallest numbers were reported in the human body louse, *P. humanus*, while the largest is seen in the mosquito *Culex pipiens* [20].

For the GST enzymes, a total of eight classes are known [19]. The Delta and Epsilon classes are unique to insects and appear to have important roles in xenobiotic detoxification [19], [41]. The other classes have known roles in other processes (see below). In the *B*. *dorsalis* transcriptome, 27 (almost 64%) of the GSTs identified pertain to the Delta and Epsilon classes and these, of course, may have similar roles in resistance. Among the other classes of GSTs, for example the omega class, the numbers found here in *B*. *dorsalis* are larger than those from *D. melanogaster*, *An. gambiae*, *A. mellifera*, *Acyrthosiphon pisum*, and *Myzus persicae* [41], [42]. This class of GSTs is also found in mammals, and is known to have proline-rich N-terminal extensions and high levels of activity with 1-chloro-2,4 dinitrobenzene (CDNB). This compound is often referred to as the universal GST substrate [43]. For the sigma class of GSTs, which appear to play roles in the flight muscle operating under oxidative stress [41], the numbers in *B*. *dorsalis* are less than those of the *A. mellifera*, *Ac. pisum*, and *My. persicae*, but more than those of *D*. *melanogaster* and *An*. *gambiae* [41], [42]. Another group of proteins appear to belong to the theta class of GSTs. This class was often originally overlooked because of the lack of affinity to GSH matrices and the lack of activity with CDNB [43]. Finally, the microsomal class of GSTs is designated as a group of membrane-associated proteins involved in eicosanoid and glutathione metabolism (MAPEG) [43]. The apparent presence of three genes in this class in *B*. *dorsalis* is also a larger number than that found in other insect species [41], [42]. These GSTs can play a role similar to that of the cytosolic enzymes in general detoxification reactions and in protection against oxidative stress [41].

For the P450 gene superfamily, overall it is large and highly diverse amongst different organisms. For example, the human genome appears to contain 57 P450 genes (along with 58 pseudogenes), distributed amongst 18 CYP families, while the mouse genome has 102 genes along with nearly 90 pseudogenes [20]. In *D. melanogaster*, there are 85 P450 genes and five pseudogenes [44]. In our study, a total of 90 P450 type genes were identified in *B*. *dorsalis*. This number is similar to the number from different *Drosophila* species (76–91), and is slightly less than that of mosquito species which range at least from 105–180 [20]. Also, because the expression of P450 genes is very diverse and is often stage specific [45], additional P450 s may be expected when transcriptome material from different developmental stages from any species are studies, including *B. dorsalis*.

The *B*. *dorsalis* P450 genes identified here can be divided into 15 families, which in turn can be placed into four major clades including the CYP2, CYP3, CYP4 and mitochondrial [44] versions. The largest family is cyp 6 (belonging to clade 3), followed by the cyp 4 family in clade 4. The families known to be involved in insecticide resistance are cyp 4, 6, 9 and 12 [46]. In *B*. *dorsalis*, a total of 66 genes identified appear to belong to these families, and therefore are also good candidates for having important roles in resistance phenomena. The number of genes in the mitochondrial clade is only 4. This is greater than the number in *My. persicae* [42] but fewer than those in other known species where genome analysis has been done [41], [42].

The carboxylesterases (COE) can be divided into 13 clades [19], one of which includes the acetylcholinesterase (AChE) enzyme. These clades can in turn be organized into three classes consisting of enzymes with dietary, pheromone and hormone processing, and neurodevelopmental functions. In *B*. *dorsalis*, a total of 37 putative carboxylesterase genes can be found, and this compares with the numbers of 34, 31, 24, 29, and 22 identified in *D. melongaster, An*. *gambiae*, *A. mellifera*, *Ac. pisum* and *My. persicae*, respectively [41], [42].

A total of 24 of the *B. dorsalis* COE genes can be placed into the same 13 clades. In *B. dorsalis*, larger numbers of COE genes appear to belong to the dietary class compared to any of the other insect species. This class is involved in the detoxification of xenobiotics. Only one of the COEs in *B*. *dorsalis* appears to belong to the JHE class, and this number is much lower than that for other insect species [41], [42]. Four of the COEs (including one AChE) in *B. dorsalis* also appear to belong to the neurodevelopmental class.

### Discovery of genes with target sites for insecticides

As shown in Table 7, Table 8, and Table 9, a total of four types of target-site sequences related to different classes of insecticides were identified using the tblastx program for genes from the closest related species in the NCBI database (identity >90%). Using this program only one isotig of 2,402 bps in length was identified as containing the *ace* gene, and it showed 98% similarity with a gene cloned and characterized from the same *B*. *dorsalis* line used here [9]. One isotig was annotated as containing a VGSC gene Domain II S4, based on a 518 bp sequence identified by comparison to the sequences from *B. oleae*. However, in insects the total length of this gene is expected to be about 6.5 Kbps (see detail in [47]). In part for this reason, the VGSG gene sequences of *Musca domestica* (X96668.1) [11] was used to search for additional isotigs. Ten were identified in this way to bring the total length to 6370 bps.

**Table 7:** Identification of *B. dorsalis* transcripts related to insecticide target site: AChE and nAChR (alpha).

Insecticide class/target site	Gene reference (bold)Isotig ID	E-value	Length	Coverage
Organophosphate, carbamates/AChE	**(AY155500, Bd) AChE (Ace)**		**2089**	
	k31_Locus_8734.2	0	2402	23.56
Neonicotinoids, spinosad/nAChR	**(EU814872) nAChR alpha-1 (AlS)**		**3279**	
	k31_Locus_5634.1 (71.77%)	1E-178	1224	8.62
	**(NM_079757) nAChR alpha-1 isoform (nAcRalpha-96Aa)**		**3469**	
	k31_Locus_42117.1	7E-34	277	3.33
	k31_Locus_34025.1	5E-20	338	4.45
	**(X53583) nAChR alpha-2**		**2210**	
	k31_Locus_21995.1	1E-37	184	2.93
	k31_Locus_57686.1	2E-52	274	3.28
	**(Y15593) nAChR alpha-3**		**2838**	
	k31_Locus_71291.1	1E-23	125	2.93
	k31_Locus_16057.1	1E-78	375	0.96
	k31_Locus_57686.1	8E-24	274	3.28
	k31_Locus_14631.1	6E-49	806	2.90
	**(FR689750) nAChR alpha-3 isoform (nicra3 gene)**		**2346**	
	k31_Locus_71291.1	9E-24	125	2.93
	k31_Locus_16057.1	1E-78	375	0.96
	**(AJ272159) nAChR alpha-4**		**1991**	
	k31_Locus_24175.1	2E-54	460	8.15
	k31_Locus_71291.1	4E-23	125	2.93
	k31_Locus_16057.1	1E-83	375	0.96
	k31_Locus_14631.1	2E-106	806	2.90
	**(AF272778) nAChR alpha-5**		**2907**	
	k31_Locus_30916.1	3E-43	211	1.71
	k31_Locus_72836.1	5E-34	190	2.37
	**(AJ554209) nAChR alpha-6**		**1665**	
	k31_Locus_58513.1	2E-39	213	1.78
	k25_Locus_61846.1	1E-32	277	2.55
	k31_Locus_61653.1	4E-07	146	3.23
	**(AJ554210) nAChR alpha-7**		**1683**	
	k31_Locus_54741.1	2E-46	277	0.61

The identities from the tblastx alignments were >90% unless noted in parentheses. Most of the gene references belong to *Drosophila melanogaster* except as listed (Bd = *Bactrocera dorsalis*).

**Table 8:** Identification of *B. dorsalis* transcripts related to insecticide target site: nAChR (beta) and Voltage-gated sodium channel.

Insecticide class/target site	Gene reference (bolded)Isotig ID	E-value	Length	Coverage
Neonicotinoids, spinosad/nAChR	**(X04016) nAChR beta-1 (ARD)**		**2353**	
	k31_Locus_3499.4	0	1482	4.01
	**(NM_079203) nAChR beta-1**		**2445**	
	k31_Locus_3499.4	0	1482	4.01
	**(X55676) nAChR beta-2**		**1714**	
	comp41437_c0_seq1	3E-98	542	4.31
	k31_Locus_59372.1	2E-52	263	2.40
	**(AJ318761) nAChR beta-3 subunit (nAcRbeta-21C gene)**		**1334**	
	k31_Locus_8600.1 (58.33%)	1E-90	1278	29.15
	**(AY005148) nAChR beta-3 subunit**		**1421**	
	k31_Locus_8600.1 (58.33%)	1E-90	1278	29.15
Pyrethroids, pyrethrins/Voltage-gated sodium channel	**(EU253453, Bo) VGSC, partial cds**		**339**	
	k33_Locus_11255.2	2E-56	543	9.77
	**(X96668) VGSC**		**6899**	
	k31_Locus_13036.1	0.004	499	9.97
	k31_Locus_19391.1	1E-53	437	3.91
	k31_Locus_25824.1	2E-74	404	5.35
	k31_Locus_34834.1	1E-18	236	7.14
	k31_Locus_6243.1	2E-173	956	9.23
	k33_Locus_11255.2	5E-51	543	9.77
	k31_Locus_10091.3	7E-32	685	7.62
	k31_Locus_24850.1	7E-19	265	3.40
	k31_Locus_13117.3	4E-120	822	9.72
	k31_Locus_19552.1	0	1523	24.76

The identities from the tblastx alignments were >90% unless noted in parentheses. Most of the gene references belong to *Drosophila melanogaster* except as listed (Bo = *Bactrocera oleae*).

**Table 9:** Identification of *B. dorsalis* transcripts related to insecticide target site: gamma-aminobutyric acid (GABA)-regulated chloride channel.

Insecticide class/target site	Gene reference (bolded) Isotig ID	E-value	Length	Coverage
Cyclodiene, fipronil/ gamma-aminobutyric acid (GABA)-regulated chloride channel	**(NP_523991) Rdl-PA,-PB,-PC**		**1821**	
	k31_Locus_44016.1	6 E-95	430	3.77
	k31_Locus_55435.1	7E-61	492	3.82
	k31_Locus_55435.2	1E-16	325	2.18
	k31_Locus_6648.1	3E-33	309	5.38
	(NP_524131) Grd		2061	
	k31_Locus_58416.1	1E-08	1\87	1.93
	k31_Locus_44016.1	2E-30	430	3.77
	k31_Locus_55435.1	1E-11	492	3.82
	k31_Locus_10782.1	2E-23	1616	16.98
	**(NP_996469) Lcch3**		**1491**	
	k31_Locus_58416.1	7E-30	187	1.93
	k31_Locus_75317.1	6E-17	123	2.20

The identities from the tblastx alignments were >90% unless noted in parentheses. The gene references belong to *Drosophila melanogaster*.

The nAChRs represent a diverse family of cys-loop ligand-gated ion channels. In contrast to the case for many animals, insects are thought to have relatively few (on the order of 10 to 12) nAChR type receptor gene families [13]. For example, *D. melanogaster* and *A. gambiae* have 10 such gene families [12], [48] while 12 are found in *B. mori* [49] and *T. castaneum* [50]. Within these families, the number of alpha subunits which contain the YxCC motif involved in nAChRs binding [51] ranges from 7 to 9, while the beta numbers typically range from 1 to 3 [12], [13], [48], [49], [50]. In *B*. *dorsalis*, 7 alpha and 3 beta subunits have also been identified as nAChRs. Each of the genes encoding these subunits are also found in anywhere from 1 to 4 isotigs. Of these, three have sequences of more than 1,000 bps in length. Also at k31_Locus_57686.1, the alpha 2 and 3 subunits are found within the same isotig. However, most of the nAChRs sequences identified here contain partial sequences, and this does limit the ability to use the YxCC motifs to infer subunit attributes. In addition in all of these cases, alternative splicing and A-to I RNA editing may also be a source of variation, and this in turn could also affect the ability to make subunit identifications [52].

Finally, in general, the GABA receptors also belong to the super family of cys-loop neurotransmitter receptors. Insect GABA receptors are divided into three classes. These are known to be encoded by either the rdl (resistance to dieldrin), grd (GABA and glycine-like receptor of *Drosophila*), or lcch3 (ligand-gated chloride channel homologue) genes (detail see Hosie et al. 1997 [53]). Alterations of the rdl gene are known to cause resistance to dieldrin and fipronil [16], [17]. Four isotigs with a total length of 1,556 bps were identified containing sequences representing rdl genes, so it seems that these classes of genes are also present in *B. dorsalis*. All the assembled sequences relating to genes with target sites for insecticides are also available at our web site, http://www.csbb.ntu.edu.tw/Bd/.

### PCR Validation

**Figure 5:**
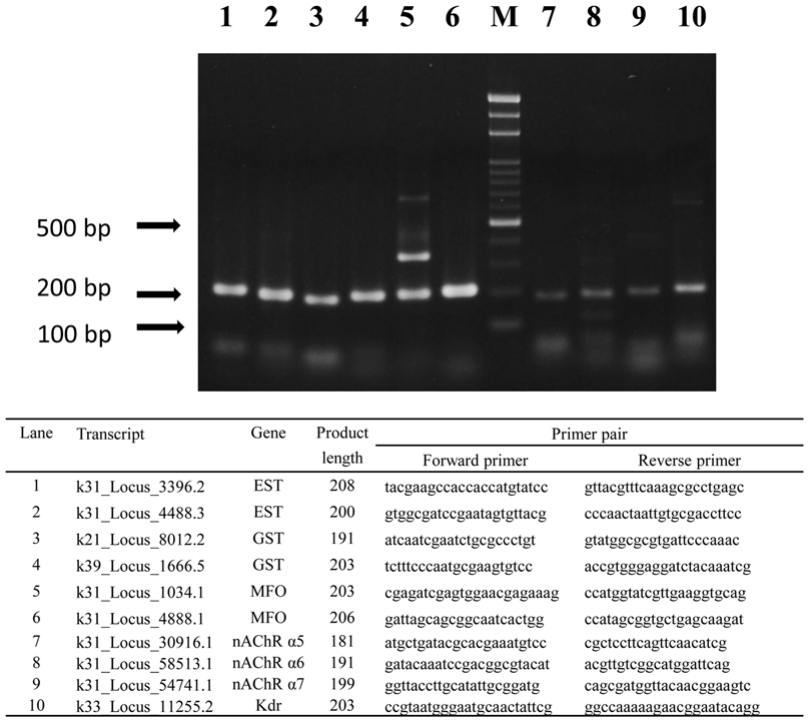
Results of PCR validation for selected sequences.

A series of genes putatively identified from the k = 31 analysis were chosen for PCR validation studies. This included a total of four genes containing potential insecticide target-site sequences, including three nAChRs (k31_Locus_30916.1, k31_Locus_58513.1, and k31_Locus_54741.1) and one kdr (k33_Locus_11255.2), along with a total of six genes encoding metabolic enzymes, including two MFOs (k31_Locus_1034.1 and k31_Locus_4888.1), two ESTs (k31_Locus_4488.3, and k31_Locus_3396.2) and two GSTs (k39_Locus_1666.5, and k21_Locus_8012.2). Each was predicted to produce transcripts approximately 200 bps in length. These were amplified using primer pairs designed from our assembled sequence data. All ten produced distinct amplicons of the expected length (Figure 5). Nine of the ten produced single band amplicons. The last produced more than one amplicon, however both of these were still consistent with the expected overall length of the transcript.

### Conclusions

This study represents a functional genomic analysis of an RNA-seq based transcriptome generated for *B*. *dorsalis*. This is the first transcriptome study using short reads (90 bp) for delivering quality transcript sequences on a large scale. Our results estimate that *B*. *dorsalis* contain no less than 11,073 genes with homology to *D. melanogaster*. In terms of genes actually or potentially involved in insecticide resistance phenomena, the distribution of transcripts in different functional groups of these genes in *B*. *dorsalis* is similar overall to that of *D. melanogaster*. This includes estimates that the *B*. *dorsalis* genome contains 90 P450 genes, 42 GST genes, and 37 COE genes. These sequence motifs were analyzed to characterize the putative polypeptide translational products and associate them with protein functions. In addition, 36 isotigs, which potentially represent 15 genes, were discovered to have potential target sites related to four different classes of insecticide resistance. This resource is believed to provide essential information to facilitate identification of genes involved in insecticide resistance, and may be of great help in designing new chemicals, compounds or other strategies for the control of this and other devastating Tephritid pest species in the future.

## Materials and Methods

### Sample preparation

The flies used in this study were from a strain originally collected in Taichung, Taiwan, and have been reared in an incubator at 24±2°C with a photoperiod of 12∶12 (L∶D) h since 1994. The newly emerged adults, maintained at a density of 500–2000 flies in each cage (39 by 19 by 6 cm), were provided with water and a standard laboratory diet consisted of a mixture of 4 parts granulated sugar to 1 part peptone (Kyokuto Seiyaku, Tokyo, Japan).

### RNA-seq

Four day old adults of generation 150 from this line were used for total RNA extraction. RNA from 30 flies was extracted using Trizol-reagent (Invitrogen, Carlsbad, CA, USA) according to the manufacturer's instructions. Approximately 1.98 mg/µL of RNA was obtained with an OD_260/280_ ratio of 2.06. The quality of total RNA was verified on 1.2% agarose-MOPS-formaldehyde denaturing gels. After extraction, the RNA was immediately stored at −80°C until it was shipped to BGI (Beijing Genomics Institute, China) for Illumina sequencing.

Poly (A) mRNA was isolated using oligo-dT beads. First-strand cDNA was generated using random hexamer-primed reverse transcription, followed by synthesis of the second-strand cDNA. From this, RNA-seq libraries were prepared following Illumina's protocols and were sequenced on the Illumina GA II platform at the BGI.

### *De novo* assembly

**Figure 6:**
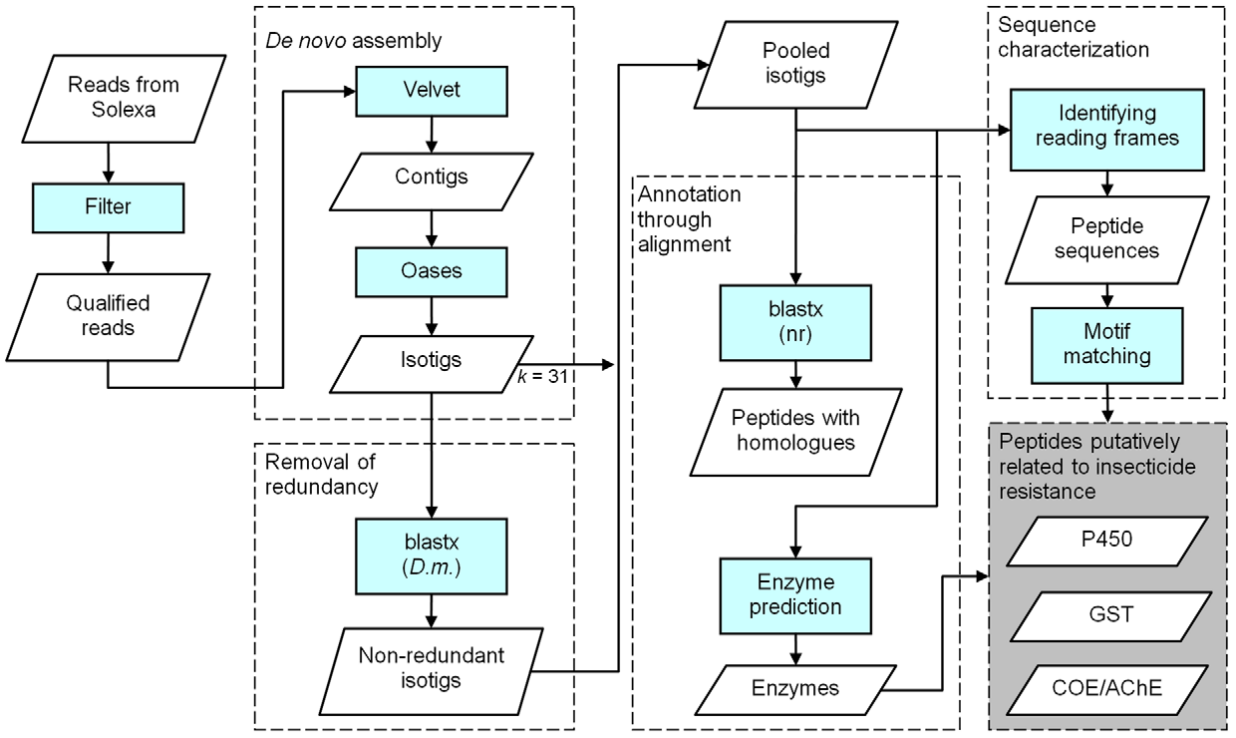
Flowchart of methods used for data analysis.

Figure 6 provides a flow diagram of the computational procedures used for this study. Details for each box in the analysis will be described in this and the following subsections. Before being assembled, the reads generated by Illumina sequencing were filtered to remove un-qualified reads as follows. First, the quality scores for reads were examined. A read was retained only if it contained at least 25 bases with quality scores >30 within the first 35 bases, starting from the 5′ end. Next, reads containing adapter sequences and one or more uncertain nucleotides (Ns) were excluded. The remaining qualified reads were fed to the short-read assembler Velvet (version: 1.0.18) [27]. The parameter *k* (the length of short words (*k*-mers) used in constructing the de Bruijn graph) was set to different odd numbers ranging from 21 to 41. This was done because recent studies [28], [29] have suggested there might not be a single optimal value for *k* for transcriptome assembly. Finally, the program Oases (http://www.ebi.ac.uk/~zerbino/oases/, version: 0.1.18) was invoked to generate isotigs from the contigs produced by Velvet.

The isotigs generated by the different settings of *k* were aligned against the protein sequences of *D. melanogaster* (19,684 RefSeq) using the blast× [30] program. The set of isotigs produced by *k* = 31 was adopted as the basis of the assembled sequence regardless, of whether or not a homologue in *D. melanogaster* was found. Homology is defined here as an alignment containing >60 amino acids and with an E-value <10^−5^. Isotigs from other settings of *k* were further considered only if found to contain homology to *D. melanogaster* proteins that were not identified in the screening of isotigs using *k* = 31.

### Transcript annotation

Non-redundant isotigs from multiple settings of *k* (as above) were pooled with the isotigs generated by setting *k* = 31. The merged set of assembled sequences was aligned against the non-redundant (nr) protein sequence database from NCBI (release: Januaray 26, 2011). Individual sequences were named based on the homologues found in the nr database meeting the minimum requirements of an alignment containing >60 amino acids and with an E-value <10^−5^.

### Enzyme prediction

The procedure for enzyme prediction was based primarily on the use of blastx [30] to align the assembled sequences against the enzyme sequences available from the Uniprot database. An isotig was assigned to an EC group if it contained a potential homologue within that group using the definition of homology described previously. We also made enzyme predictions using pattern matching for sequence signatures. Here, enzyme sequences that met the homology criteria and were well annotated in the Uniprot database were downloaded. Also, in several cases, an isotig was assigned to more than one EC group by different E-values without a clear ranking as a first priority. In these cases the motif analysis method described in the following section would be invoked to confirm the EC predictions.

### Motif analysis

Assembled sequences were further characterized based on E1DS motifs [36] derived using the following procedures. Different sequences relating to the same EC number were grouped, and mining for a sequential pattern was conducted for motif discovery using WildSpan [38]. Before motif discovery the isotigs were translated using six all possible reading frames to generate peptide sequences, all of which were analyzed. The performance of WildSpan to make motif predictions of enzyme functions and catalytic sites has been evaluated in E1DS. It has also been shown [38] that the accuracy of these predictions using WildSpan motifs is higher than that using PROSITE patterns [54], both in terms of sensitivity and specificity rates. On the other hand, using predictions from sequence alignments, PSI-BLAST [30] has been shown to have a higher sensitivity rate for enzyme prediction compared to the use of E1DS motifs, although with a lower specificity rate [36].

### GO analysis

For the analysis of the distribution of transcripts in different functional groups, GO analysis was conducted by using BiNGO (version 2.42) [35] to obtain associated values based first on the broad headings of ‘molecular function’, ‘biological process’ and ‘cell component’ as used in the characterization of *D. melanogaster*. Associated GO terms were also derived for the 8,815 NCBI's Entrez GeneIDs as suggested by BiNGO. These transcripts were ultimately classified into one of either the 13 major GO categories under ‘molecular function’ [25], the 14 major categories under ‘biological process’ [26] or the 15 major categories (in this case limited to those with more than 600 genes) under ‘cell component’. In addition, a transcript could be split among different GO categories since it might be annotated as being within more than one category. Further comparisons of the distribution of transcripts in different functional groups between *B. dorsalis* (8,815 GeneIDs), *C. capitata* (4,810 GeneIDs) and *D. melanogaster* (14,904 GeneIDs) were developed by applying the same procedure.

### PCR Validation

A total of ten transcripts, four of which were related to various insecticide target site sequences and six of which were related to three main metabolic enzymes, were chosen for validation by PCR assays. Specific primer pairs for each transcript were designed using eprimer 3 (from the program EMBOSS Explorer). For the assay, from one picogram up to 5 µg of total RNA was used for the first strand synthesis of cDNA in a 20 µl total volume reaction mix using the SuperScript II reverse transcription cDNA synthesis system (Invitrogen Inc.) according to the manufacturer's instructions. A poly dT (20) oligo was used as the reverse primer. Each specific primer pair was used to amplify the various cDNA fragments using a cycling profile consisting of an initial denaturation at 94°C for 3 min followed by 35 cycles consisting of 94°C for 30 s, 60°C for 20 s and 72°C for 30 s. A final extension was also done at 72°C for 10 min.

## Supporting Information

Figure S1 **Sample E1DS motifs.**
(DOC)

Table S1 **Assessment of assembly quality by alignment of assembled sequences with known complete coding sequences from *Bactrocera dorsalis.***
(DOC)

Table S2 **Sample alignments of long isotigs with putative homologues.**
(DOC)

Table S3 **Predicted P450-related transcripts (EC 1.14.14.1).**
(XLS)

Table S4 **Predicted GST-related transcripts (EC 2.5.1.18).**
(XLS)

Table S5 **Predicted COE-related transcripts (EC 3.1.1.1).**
(XLS)

Table S6 **Predicted AChE-related transcripts (EC 3.1.1.7).**
(XLS)
