# Supplementary material for: Discovery of Genes Related to Insecticide Resistance in Bactrocera dorsalis by Functional Genomic Analysis of a De Novo Assembled Transcriptome
Source: PLoS One. 2012 Aug 7;7(8):e40950. doi: 10.1371/journal.pone.0040950 (PMC3413685; doi:10.1371/journal.pone.0040950)

**Figure S1** – **Sample E1DS motifs.** The example protein shown here is plotted in *strand* presentation. Two motif fragments are highlighted using green and yellow colors.


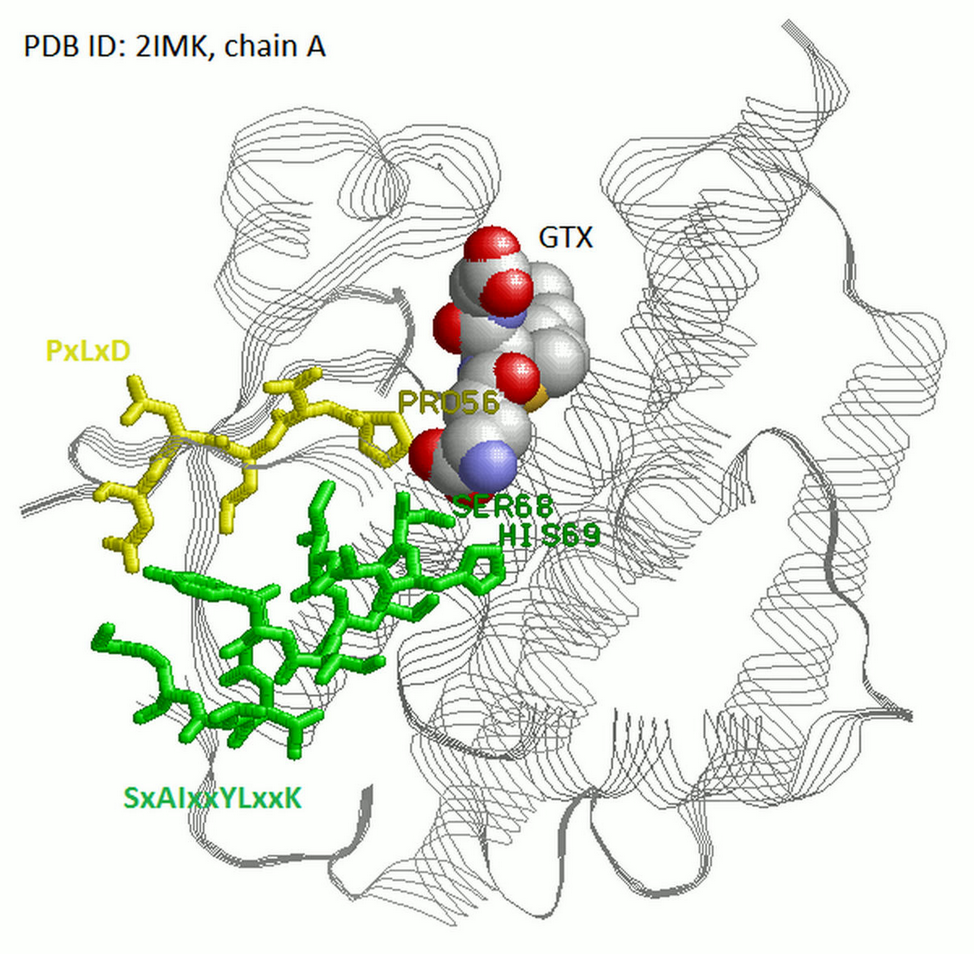

Supplement: Figure S1 — Sample E1DS motifs. (DOC) [file pone.0040950.s001.doc]
