# Supplementary material for: Discovery of Genes Related to Insecticide Resistance in Bactrocera dorsalis by Functional Genomic Analysis of a De Novo Assembled Transcriptome
Source: PLoS One. 2012 Aug 7;7(8):e40950. doi: 10.1371/journal.pone.0040950 (PMC3413685; doi:10.1371/journal.pone.0040950)
Supplement: Table S1 — Assessment of assembly quality by alignment of assembled sequences with known complete coding sequences from Bactrocera dorsalis. (DOC) [file pone.0040950.s002.doc]

1. – Assessment of assembly quality by alignment of sequences with known complete coding sequences from *Bactrocera dorsalis* genes

| **Reference name (shaded)**  **Isotig ID** | **Start^a^** | **End^b^** | **Identity (%)** | **Alignment length (bp)** | **Length (bp)** | **Coverage^c^** |
| --- | --- | --- | --- | --- | --- | --- |
| (GU591409) heat shock protein 70 BD2 |  |  |  |  | 2263 |  |
| k31_Locus_3628.1 | 63 | 2139 | 97.83 | 2077 | 2077 | 15.51 |
| (GU591408) heat shock protein 70 BD1 |  |  |  |  | 2086 |  |
| k31_Locus_708.7 | 185 | 527 | 99.71 | 343 | 343 | 390.44 |
| k31_Locus_6606.2 | 836 | 1115 | 99.64 | 280 | 280 | 321.28 |
| k31_Locus_913.2 | 1414 | 1663 | 98.4 | 250 | 250 | 200.30 |
| k31_Locus_17715.1 | 1811 | 2065 | 99.22 | 255 | 281 | 514.23 |
| (FJ167395) target of rapamycin |  |  |  |  | 8060 |  |
| k31_Locus_69128.1 | 643 | 887 | 99.59 | 245 | 245 | 3.24 |
| k31_Locus_47731.1 | 1122 | 1250 | 100 | 129 | 130 | 0.69 |
| k31_Locus_61783.1 | 1641 | 1767 | 99.21 | 127 | 127 | 2.28 |
| k31_Locus_17065.1 | 2582 | 2793 | 100 | 212 | 212 | 3.19 |
| k31_Locus_70682.1 | 2961 | 3094 | 99.25 | 134 | 134 | 1.34 |
| k31_Locus_48106.1 | 3492 | 3665 | 98.28 | 174 | 174 | 2.74 |
| k31_Locus_41471.1 | 4737 | 4859 | 100 | 123 | 123 | 2.20 |
| k31_Locus_41472.1 | 4898 | 5098 | 99.5 | 201 | 201 | 1.79 |
| k31_Locus_34850.1 | 5497 | 5630 | 96.27 | 134 | 135 | 2.00 |
| k31_Locus_61614.1 | 6151 | 6395 | 98.37 | 245 | 245 | 3.42 |
| k31_Locus_36985.1 | 6468 | 6766 | 99.33 | 299 | 299 | 2.41 |
| k31_Locus_30736.1 | 6895 | 7249 | 99.44 | 355 | 355 | 3.71 |
| k31_Locus_34414.1 | 7769 | 7897 | 100 | 129 | 129 | 2.09 |
| (HM195185) ultraspiracle protein |  |  |  |  | 1782 |  |
| k31_Locus_13072.1 | 277 | 1460 | 99.83 | 1184 | 1426 | 9.18 |
| (AY125819) guanylyl cyclase receptor |  |  |  |  | 4673 |  |
| k31_Locus_73528.1 | 107 | 227 | 96.69 | 121 | 121 | 1.49 |
| k31_Locus_33682.1 | 899 | 1141 | 99.18 | 243 | 243 | 3.53 |
| (AF368054) vitellogenin 2 precursor |  |  |  |  | 1434 |  |
| k31_Locus_23853.1 | 14 | 156 | 99.3 | 143 | 143 | 0.91 |
| k31_Locus_30623.1 | 644 | 1082 | 99.32 | 439 | 439 | 1.23 |
| (AF368053) vitellogenin 1 precursor |  |  |  |  | 1507 |  |
| k31_Locus_335.4 | 151 | 1417 | 99.37 | 1267 | 1388 | 36.63 |
| (FJ416628) guanylyl cyclase receptor beta |  |  |  |  | 3645 |  |
| k31_Locus_33682.1 | 455 | 697 | 99.18 | 243 | 243 | 3.53 |
| (AY155500) acetylcholinesterase (Ace) |  |  |  |  | 2209 |  |
| k31_Locus_8734.2 | 189 | 2209 | 98.09 | 2044 | 2424 | 13.00 |

**Table S1 – Assessment of assembly quality by alignment of sequences with known complete coding sequences from *Bactrocera dorsalis* genes (continued)**

| **Reference name (shaded)**  **Isotig ID** | **Start^a^** | **End^b^** | **Identity (%)** | **Alignment length (bp)** | **Length (bp)** | **Coverage^c^** |
| --- | --- | --- | --- | --- | --- | --- |
| (AY324653) soluble guanylyl cyclase 1 |  |  |  |  | 2680 |  |
| k31_Locus_7727.2 | 58 | 2648 | 98.27 | 2595 | 2595 | 17.47 |
| (EU621792) odorant receptor Or83b (Or83b) |  |  |  |  | 1422 |  |
| k31_Locus_37153.1 | 762 | 991 | 99.13 | 230 | 230 | 2.49 |
| (EU564816) odorant-binding protein (OBP) |  |  |  |  | 757 |  |
| k31_Locus_4355.1 | 5 | 685 | 95.45 | 681 | 692 | 34.79 |
| (AY669317) male-specific double sex protein (dsx-M) |  |  |  |  | 2250 |  |
| k31_Locus_11208.2 | 519 | 1432 | 97.54 | 934 | 934 | 8.19 |
| k31_Locus_11208.1 | 1031 | 1432 | 99.75 | 402 | 402 | 4.70 |
| k31_Locus_38968.1 | 1462 | 1739 | 97.54 | 284 | 284 | 2.31 |
| (AY575956) opsin Rh1 (Rh1) |  |  |  |  | 1593 |  |
| k31_Locus_688.6 | 239 | 785 | 99.45 | 549 | 549 | 588.45 |

^a^ the start position of the alignment on the reference

^b^ the end position of the alignment on the reference

^c^ the average depth of the isotig
