# Supplementary material for: Discovery of Genes Related to Insecticide Resistance in Bactrocera dorsalis by Functional Genomic Analysis of a De Novo Assembled Transcriptome
Source: PLoS One. 2012 Aug 7;7(8):e40950. doi: 10.1371/journal.pone.0040950 (PMC3413685; doi:10.1371/journal.pone.0040950)
Supplement: Table S2 — Sample alignments of long isotigs with putative homologues. (DOC) [file pone.0040950.s003.doc]

**Table S2 – Sample alignments of long isotigs with putative homologues**

| **Isotig ID** | **Length in bps** | **Identity (%)** | **Alignment length in amino acids** | **Reference** | **Name of the reference** |
| --- | --- | --- | --- | --- | --- |
| k31_Locus_1409.1 | 15706 | 90.88 | 5134 | NP_476991.1 | ryanodine receptor 44F, isoform A |
| k31_Locus_1409.5 | 11185 | 91.87 | 3643 | NP_476993.1 | ryanodine receptor 44F, isoform C |
| k31_Locus_3171.4 | 10665 | 42.3 | 2889 | NP_788624.1 | molecule interacting with CasL, isoform F |
| k31_Locus_443.8 | 12745 | 71.3 | 4234 | NP_725339.1 | short stop, isoform H |
| k31_Locus_395.43 | 10535 | 77.34 | 3199 | NP_001027224.2 | Muscle-specific protein 300, isoform B |
| k31_Locus_903.2 | 10240 | 66.32 | 3055 | NP_001027222.2 | Muscle-specific protein 300, isoform D |
| k31_Locus_483.5 | 12365 | 54.56 | 4168 | NP_001162826.1 | bent, isoform G |
